# Analgesia and spread of erector spinae plane block in breast cancer surgeries: a randomized controlled trial

**DOI:** 10.1186/s12871-022-01860-w

**Published:** 2022-10-17

**Authors:** Ahmed Mohamed Mohamed Rabah Abdella, Emad Eldin Abd El Monem Arida, Nagwa Ahmed Megahed, Wessam Zakaria El-Amrawy, Walid Mohamed Ahmed Mohamed

**Affiliations:** 1 Department of Anesthesia and Pain Management, Medical Research Institute, University of Alexandria, 165 El-Horeya Rd, Al Ibrahimeyah Qebli WA Al Hadrah Bahri, Qesm Bab Sharqi, Alexandria, Egypt; 2 Department of Anesthesia and Surgical Intensive Care, Faculty of Medicine, University of Alexandria, Alexandria, Egypt; 3 Department of Diagnostic Radiology, Medical Research Institute, University of Alexandria, Alexandria, Egypt

**Keywords:** Erector spinae plane block, Ultrasound guided, Analgesia, Spread, and Breast cancer surgery

## Abstract

**Background:**

To evaluate the analgesic efficacy and spread of variable volumes of local anesthetics (LA) in Erector spinae plane block (ESPB).

**Methods:**

Sixty patients aged between 18 and 50 years with an ASA I-II and scheduled for breast cancer surgery were randomized to receive either ESPB with 20 ml 0.25% bupivacaine (Standard volume ESPB), or with 40 ml 0.125% bupivacaine (High volume ESPB), or no ESPB (GA only group). The primary outcome was pain intensity evaluated by the visual analogue scale (VAS), 12 hours after surgery. *P*-values < 0.05 were considered the cutoff point for statistical significance. The secondary outcomes were pain at rest and pain on movement evaluated by the VAS, craniocaudal injectate spread, to paravertebral (PV) and epidural spaces assessed by CT, clinical dermatomal spread, level of sedation or agitation, and patient satisfaction with anesthesia and analgesia.

**Results:**

VAS at rest 12 h after surgery was less in both intervention groups compared to the control (1.75 ± 0.79 vs. 1.6 ± 0.88 vs. 3.4 ± 1.96, *p* = 0.001). The LA had extended further in the high volume group than the standard volume group (11.20 ± 3.07 vs. 9.15 ± 2.54 vertebral levels, *p* = 0.027). No difference of the spread to PV or epidural spaces between the 2 intervention groups. More dermatomes were covered in the high volume group (7.20 ± 2.12 vs. 5.75 ± 1.37 dermatomes, *p* = 0.014). Agitation was higher in the GA only group than both ESPB groups in the first 8 postoperative hours. Patients were more satisfied in both ESPB groups than the GA only group.

**Conclusions:**

Preoperative ESPB is an excellent analgesic modality and it can also attenuate both postoperative agitation and sedation. Doubling the injectate volume enhances the craniocaudal spreading and may be useful for surgeries requiring multiple dermatomes. However, larger volume has no effect on analgesic efficacy or patient satisfaction as there is no further spread to the PV, epidural spaces or spinal nerve rami.

**Trial registration:**

NCT04796363 (12/3/2021).

## Background

The most prevalent malignancy in females worldwide is breast cancer [1]. Breast cancer surgeries cause considerable acute postoperative pain which needs a comprehensive preoperative plan of multimodal analgesia including regional analgesia [2, 3]. If not adequately treated, it will increase postoperative morbidity, delay wound healing, prolong the period of hospital stay, and lead to the development of post-mastectomy pain syndrome [4, 5]. Postoperative hospital stay for breast cancer patients has shortened owing to not only the less invasive surgical techniques, but also the efficient pain relief protocols including regional anesthesia [6].

Ultrasound-guided fascial plane blocks are novel techniques which showed effectiveness in managing post-mastectomy pain [7–9]. Erector spinae plane block (ESPB) is a relatively easy to perform procedure with clearly apparent sonographic features, and a catheter could be simply introduced into the plane following injection-induced distention [10]. Its efficacy depends on the compartmental spread and the local anesthetic (LA) distribution to nearby target nerves. The LA absorption and diffusion have a role in determining ESPB quality as LA may diffuse anteriorly to the ventral and dorsal rami of the spinal nerves and through the inter-transverse connective tissue to enter the thoracic paravertebral (PV) space [11].

As the erector spinae (ES) fascia extends from the nuchal fascia cranially to the sacrum caudally, LA agents extend through several levels and the block can be effective over a large area [12]. LA volume and concentration are important factors for ESPB with volumes ranging from 10 to 40 mL have been used [13]. However, the optimum volume, concentration, distribution and dermatomal coverage are still undetermined [14].

Most studies exploring the mechanism of action of ESPB were cadaveric studies which had conflicting results, as not all of them demonstrated extensive dye spread [15, 16]. Moreover, the cadaveric models failed to explain the relation between LA volume, analgesic efficacy and the LA spread as they had many limitations. The biomechanical characteristics of cadaveric tissues differ significantly from those of the living tissues, and injected fluids could not diffuse evenly over all tissue planes. There is no consensus on whether an embalmed or fresh frozen cadaver model is best for examining the physical dissemination of injected fluid. Methylene blue is the most often used corpse dye; however it has been criticized for its alleged tendency to spread too extensively. Inadequate dissection technique could also have contributed to dye spreading in patterns that might not occur in intact fascial spaces [17].

Therefore, the primary aim of our study is to evaluate the effect of ultrasound guided (USG) ESPB using different LA volumes on analgesic efficacy in breast cancer surgery patients by exploring the radiological LA spread and the clinical dermatomal coverage. Our hypothesis is that a preoperative, high volume ESPB leads to better postoperative analgesia, less agitation, less sedation and higher levels of satisfaction due to the more extensive LA spread than the standard volume.

## Methods

### Study design

- This study was designed after the Institutional Review Board approval (IRB NO: 00012098), Faculty of Medicine, Alexandria University, Egypt (chairperson of ethics committee Prof. Dr. Maha Ghanem) on November 19, 2020, in accordance with principles of the Declaration of Helsinki (1964) and its subsequent amendments. Written informed consent form was obtained from all participants.
- The sample size was determined according to the recommendations of the department of biomedical informatics and medical statistics, Medical Research Institute using NCSS 2004 & PASS 2000 program. A minimal sample size of 20 in each group was required to achieve 90% power (beta = 0.1) and to detect a difference of 1.5 in the median visual analogue scale (VAS) between groups assuming common standard deviation of 2.5, using F test, at level of significance (alpha) = 0.05.
- Thereafter, a single center, prospective, randomized controlled, triple-blind trial was performed between March 2021 and January 2022. This trial was prospectively registered at ClinicalTrials.gov (NCT04796363; registration date: 12/3/2021).

### Patients

Eligible patients for this trial were those who were between 20 and 50 years with American society of Anesthesiology (ASA) I-II and underwent mastectomy. Exclusion criteria were a known allergy or contraindication to any of the studied medications or anesthetic agents, scoliosis or any vertebral anomalies or previous spinal surgeries, morbid obesity (Body mass index ≥40 kg/m2), chronic opioid analgesic use, pregnancy, infection at the site of injection, duration of surgery more than 90 minutes, and renal impairment.

### Randomization and blinding

Patients were randomly allocated using a computer generated random table (Graphpad Software, Inc., La Jolla, CA) and an allocation ratio of 1:1:1 was used to assign patients to receive either ESPB with 20 ml 0.25% bupivacaine (**Standard volume ESPB**), or with 40 ml 0.125% bupivacaine (**High volume ESPB**), or no ESPB (**GA only** group). Blinding of the research personnel was maintained throughout the whole observation period including all postoperative follow-ups.

In the absence of the primary anesthesia providers, a specialized regional anesthesia team performed the regional blocks for the intervention groups in the designated block room and inserted catheter in all patients.

### Intervention

All patients received intravenous (IV) midazolam (0.05 mg/kg) and fentanyl (0.5 μg/kg) 3 minutes before performance of the block. USG ESPB block was performed in the block room at Medical Research Institute hospital by the specialized regional anesthesia team, who were not included in the study. Patients were placed in the prone position and a high-frequency linear probe (L 6-12 MHz) of SonoSite, S nerve, 2 D machine, USA was prepared and covered by transparent dressing (Tegaderm®). After skin preparation, the ultrasound probe was placed 2.5–3 cm lateral to the spinous process in a parasagittal oblique plane, at the seventh cervical vertebra and moved caudally till T4.

After anatomical scanning and identification of the transverse process of T4 and the three muscles (Trapezius, Rhomboid major and Erector spinae), 2 ml of lidocaine 2% was used to numb the skin then 18-gauge Tuohy needle was advanced cranio-caudally towards the lateral border of T4 transverse process using the in-plane technique. The needle tip was located in the fascial plane between the transverse process and erector spinae muscle. The correct needle position was tested by injecting 2 ml of saline resulting in hydro-dissection of the plane followed by the injection of:

- 20 ml bupivacaine 0.25% and 5 ml of radio-contrast dye (Omnipaque) in the standard volume ESPB group.
- 40 ml bupivacaine 0.125% and 5 ml of radio-contrast dye (Omnipaque) in the high volume ESPB group.

Then, insertion of epidural catheter 2 to 3 cm over the tip of the Tuohy needle under real-time US guidance was done in the intervention groups.

In the control group, for the purpose of blinding, the skin was infiltrated by the LA and the catheter was left above the skin and similar to the intervention groups it was covered by opaque adhesive tape.

### CT assessment

Patients were kept in the prone position during transportation to the radiology department where a CT scan of the thoracic region, partially extended to the lower neck and upper abdomen, was performed 15 minutes after the block. The spread of the injected solution in the Erector spinae plane (ESP) was evaluated, and interpreted by the same radiologist, using DICOM image processing for Mac (OsirixX, PixmeoSARL; Bern, Switzerland). Three-dimensional digital reconstruction of the distribution of the injected contrast was obtained.

Points of assessment were as follow:

1. Craniocaudal spread.
2. Spread to the PV space.
3. Spread to the epidural space.
4. Exiting nerve roots.
5. Crossing of the midline.

Patients were carefully monitored during the whole transportation and during CT scanning, O2 and resuscitation equipment were readily available. Then they were returned to the operating theater and received standardized anesthetic technique. General anesthesia was induced in each group by IV fentanyl 1 μg/kg, propofol (2.5 mg/kg) and cisatracurium (0.15 mg/kg) to facilitate endotracheal intubation. Anesthesia was maintained with isoflurane (1.2 – 1.5%) and oxygen/air mixture (50, 50%). Incremental doses of cisatracurium 0.03 mg/kg were given to maintain neuromuscular blockade guided by train of four (TOF) count using the nerve stimulator module of (TOF watch –Organon-Ireland). Ventilation was maintained at a tidal volume of 6 ml/kg and a rate to adjust the end-tidal carbon dioxide at (35-40 mmHg) using the ventilator (Fabius GS- Drager-Germany). At the end of surgery, anesthesia was discontinued, residual neuromuscular block was antagonized by atropine 0.01 mg/kg and neostigmine 0.04 mg/kg, the trachea was extubated and patients were transferred to the postoperative anesthesia care unit (PACU) for the next 24 hours.

### Postoperative analgesia

Bupivacaine 50 mg, with the same volume and concentration assigned to each group, was given in the epidural catheter at the end of surgery in the standard volume and high volume ESPB groups by the specialized regional anesthesia team. In the PACU, pain score (as with other measurements) were assessed by a physician not involved in the study design.

IV morphine patient controlled analgesia (PCA) was prepared by 50 mg morphine diluted with 45 ml normal saline resulting in a concentration of 1 mg morphine /1 ml.

1. Bolus dose of 0.05 mg/kg.
2. Lockout Interval: 10 minutes.
3. four-hourly limiting dose was 10 mg.

If VAS still ≥4, patients were administered Ketorolac 30 mg IV as rescue analgesia.

### Study endpoints

The primary outcome of this RCT was pain assessment during rest at 12 h after surgery evaluated by the VAS, (0-10) where 0 = no pain, 10 = worst imaginable pain.

Secondary endpoints included:

- Craniocaudal injectate spread, spread to PV space and epidural space assessed by CT.
- Dermatomal spread was evaluated by the presence of hypoesthesia when a piece of cotton soaked in iced water was applied along the mid-clavicular line, 15 minutes after the block.
- Pain at rest, evaluated by the VAS, at 1, 2, 3, 4, 8, 16, 20 and 24 h after surgery.
- Pain with arm movement, evaluated by the VAS, at 1, 2, 3, 4, 8, 12, 16, 20 and 24 h after surgery.
- Level of agitation or sedation assessed by Richmond agitation –sedation scale (RASS).
- Patient satisfaction with anesthesia and analgesia assessed by Likert scale (self-report scale where 0 = strong dissatisfaction, 1 = dissatisfaction, 2 = neutral, 3 = satisfaction and 4 = strong satisfaction).

### Statistical analysis

Data were fed to the computer and analyzed using IBM SPSS software package version 20.0. (Armonk, NY: IBM Corp). Qualitative data were described using number and percent. The Shapiro-Wilk test was used to verify the normality of distribution. Quantitative data were described using range (minimum and maximum), mean, standard deviation and median. Significance of the obtained results was judged at the 5% level.

The used tests were:

1. Chi-square test: For categorical variables, to compare between different groups.
2. Fisher’s Exact or Monte Carlo correction: Correction for chi-square when more than 20% of the cells have expected count less than 5
3. Student t-test: For normally distributed quantitative variables, to compare between two studied groups.
4. F for One way ANOVA test: For normally distributed quantitative variables, to compare between more than two groups, and Post Hoc test (Tukey) for pairwise comparisons.
5. ANOVA with repeated measures: For normally distributed quantitative variables, to compare between more than two periods or stages, and Post Hoc test (Bonferroni adjusted) for pairwise comparisons.
6. Kruskal Wallis test: For abnormally distributed quantitative variables, to compare between more than two studied groups and Post Hoc (Dunn’s multiple comparisons test) for pairwise comparisons.
7. Friedman test: For abnormally distributed quantitative variables, to compare between more than two periods or stages and Post Hoc Test (Dunn’s) for pairwise comparisons.

## Results

**Fig. 1:**
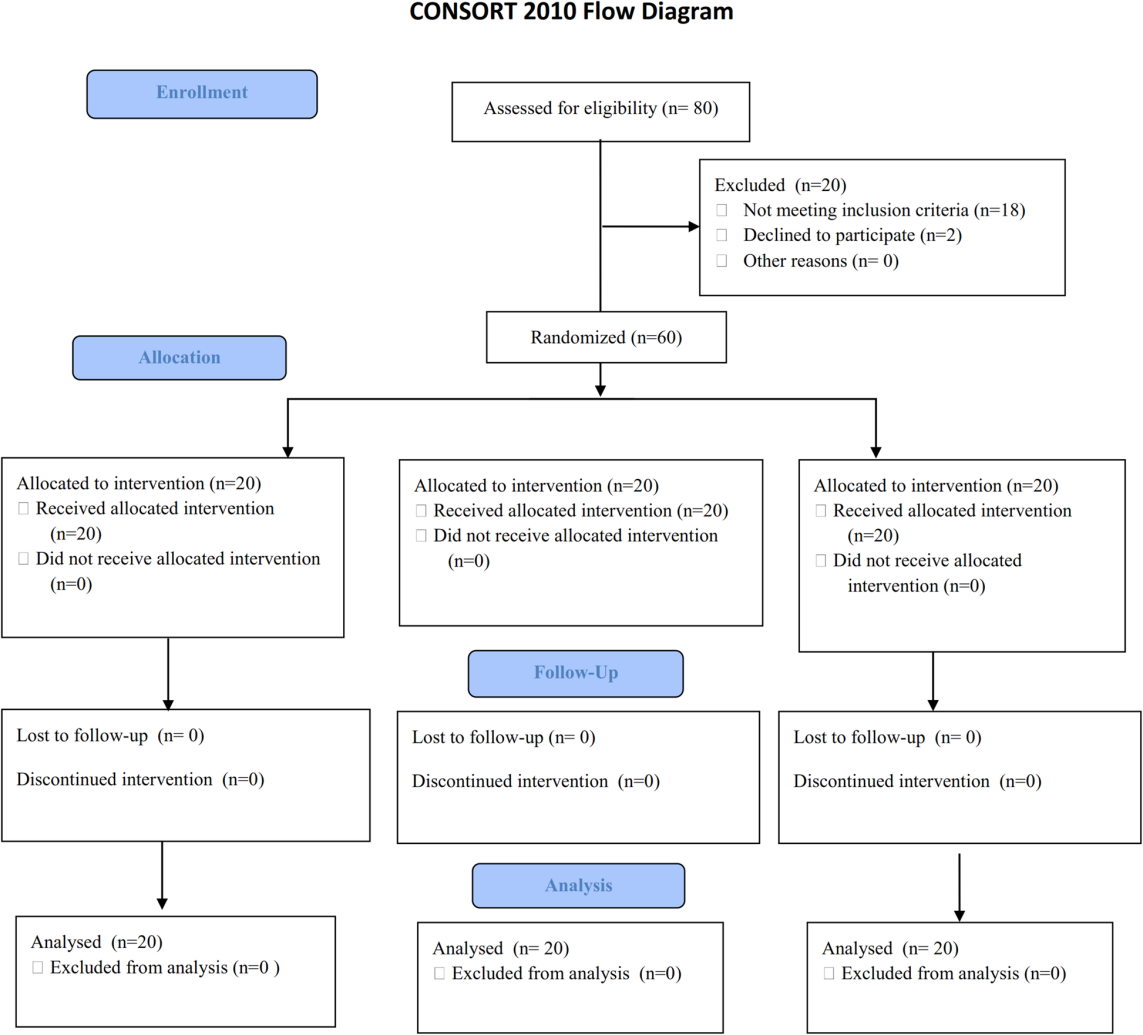
CONSORT flow diagram of the study participants

Of the 80 patients screened, 60 patients (20 per study group) were enrolled between March, 2021 and January, 2022. Flow diagram of the trial and patients’ demographics are shown in Fig. 1; Table 1, respectively. No patient dropped out of this study.

**Table 1:** Comparison among the three groups based on the demographics

Demographic data	Standard volume group (***n*** = 20)	High volume group (***n*** = 20)	GA only group (***n*** = 20)	Test of sig.	P
**Age (years)**					
Mean ± SD.	42.95 ± 7.48	41.05 ± 9.20	43.20 ± 7.15	**F =** 0.433	0.651
Median (Min. – Max.)	44.50(24.0 – 50.0)	44.0 (26.0 – 50.0)	45.0 (28.0 – 50.0)		
**Female sex**	20	20	20		
**Weight (kg)**					
Mean ± SD.	80.50 ± 10.69	79.25 ± 12.02	83.60 ± 9.45	**F =** 0.865	0.427
Median (Min. – Max.)	81.0(65.0 – 105.0)	77.50(60.0 – 100.0)	82.50(68.0 – 105.0)		
**Height (cm)** Mean ± SD	166 ± 3.5	165 ± 3.7	167 ± 3.4	**F = 1.600**	0.211
**BMI (kg / m2)**	29.2 ± 2.65	29.1 ± 3.11	29.96 ± 2.17	**F = 0.620**	0.541
**Surgery type**				χ^2^ = 0.997	^MC^p = 1.000
MRM (Modified radical mastectomy)	9	9	8		
Mastectomy without ALND (axillary lymph node dissection, eg: negative sentinel LN biopsy)	4	3	4		
Breast conservative surgery with ALND	5	6	5		
Breast conservative surgery without ALND	2	2	3		

*SD* Standard deviation, *F* F for One way ANOVA test

p: *p* value for comparing among the three groups

### Analgesia

**Fig. 2:**
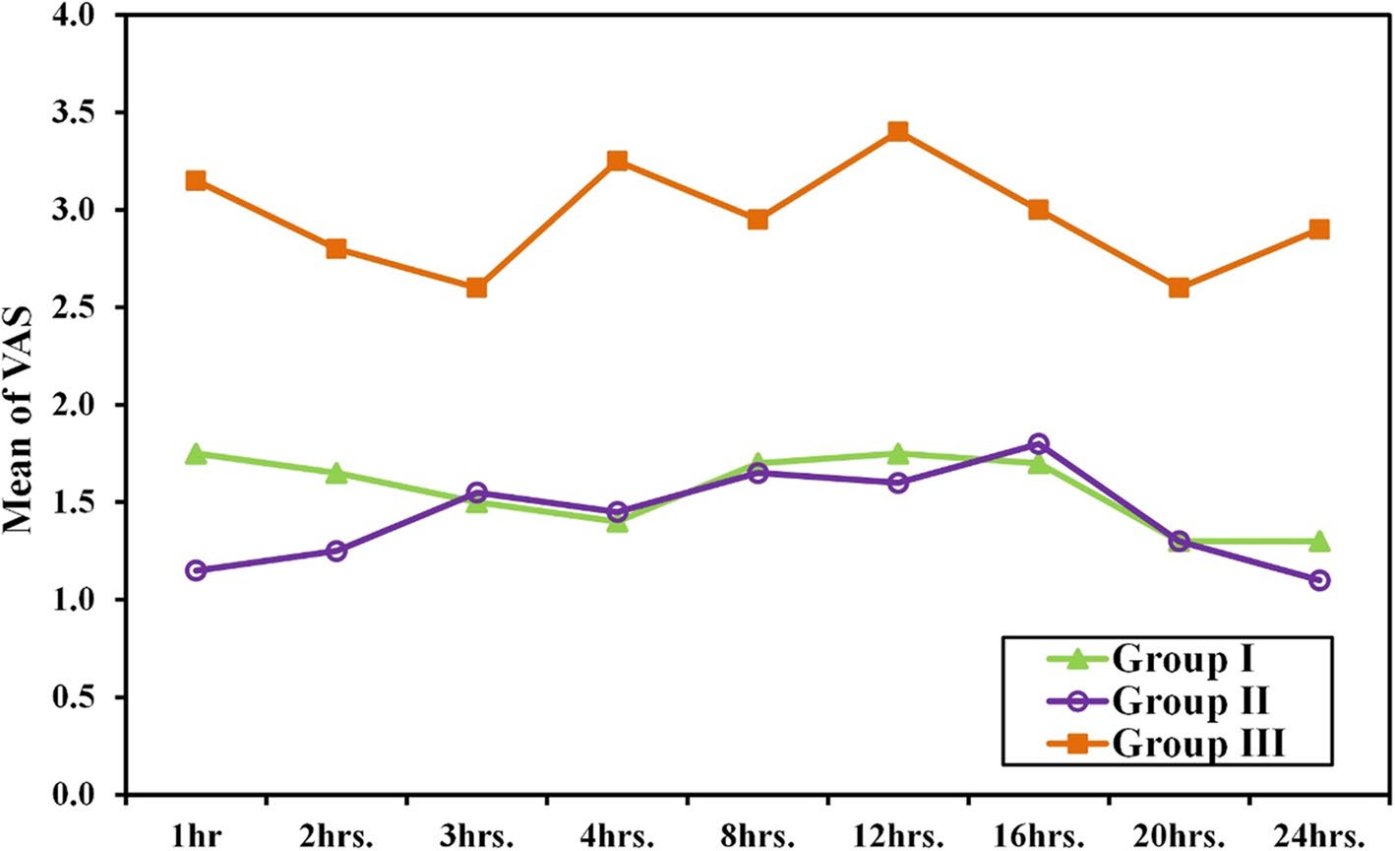
Comparison among the three groups based on VAS during rest

**Fig. 3:**
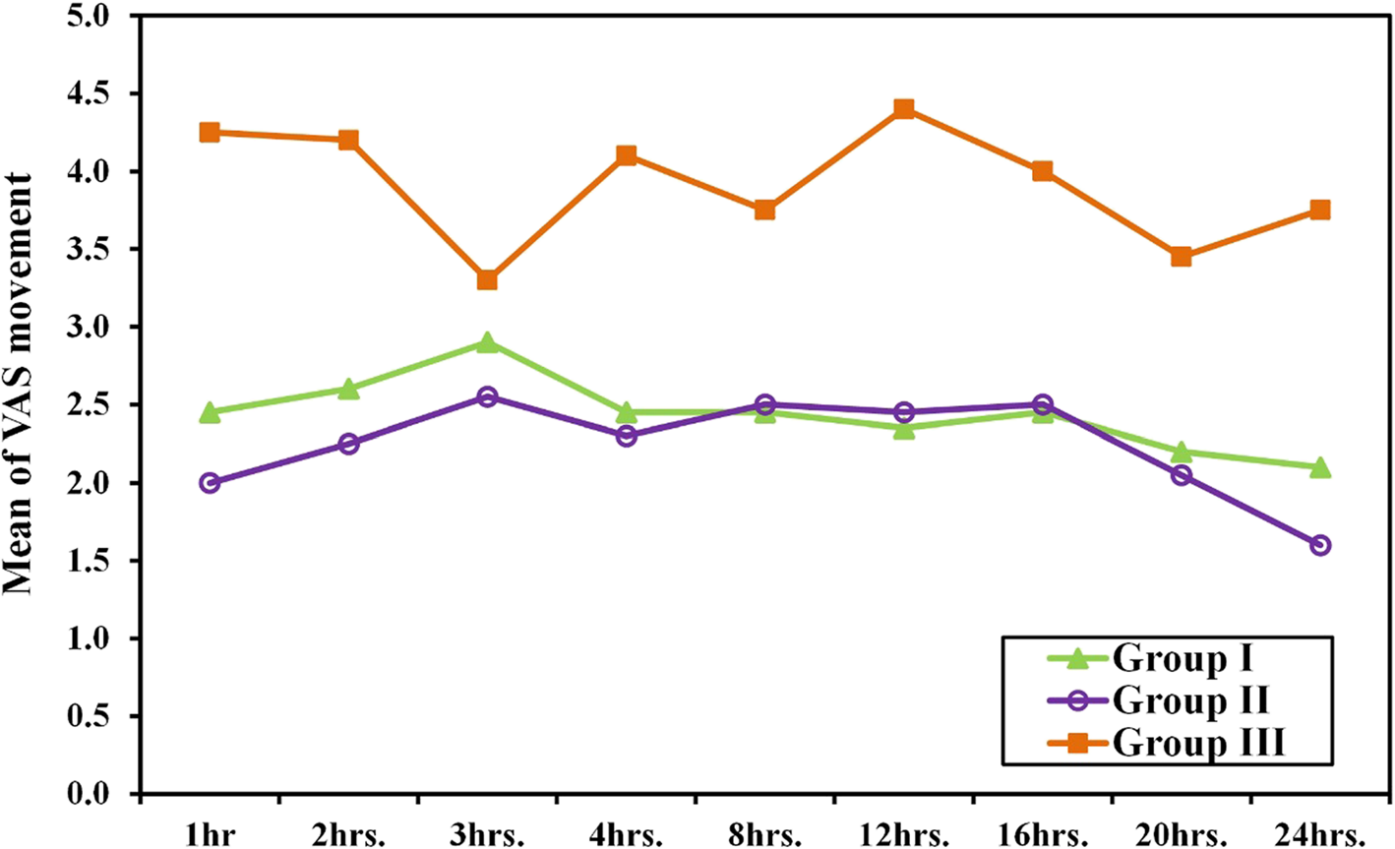
Comparison among the three groups based on VAS during arm movement

The primary and secondary analgesia outcomes are shown in Figs. 2 and 3.VAS at 12 h postoperative was less in high volume ESPB and standard volume ESPB than the GA only group (1.75 ± 0.79 vs. 1.6 ± 0.88 vs. 3.4 ± 1.96, *p* = 0.001). There were no statistically significant differences between the two intervention groups in all time points. On the other hand, VAS was less in both the high volume ESPB and the standard volume ESPB than GA only group in all time points. (Fig. 2).

VAS during arm movement changed similar to the changes recorded during rest except at 3-hour, there was no significant difference between each of the intervention groups and the control. (Fig. 3).

### Injectate spread

#### Radiological assessment by CT

**Fig. 4:**
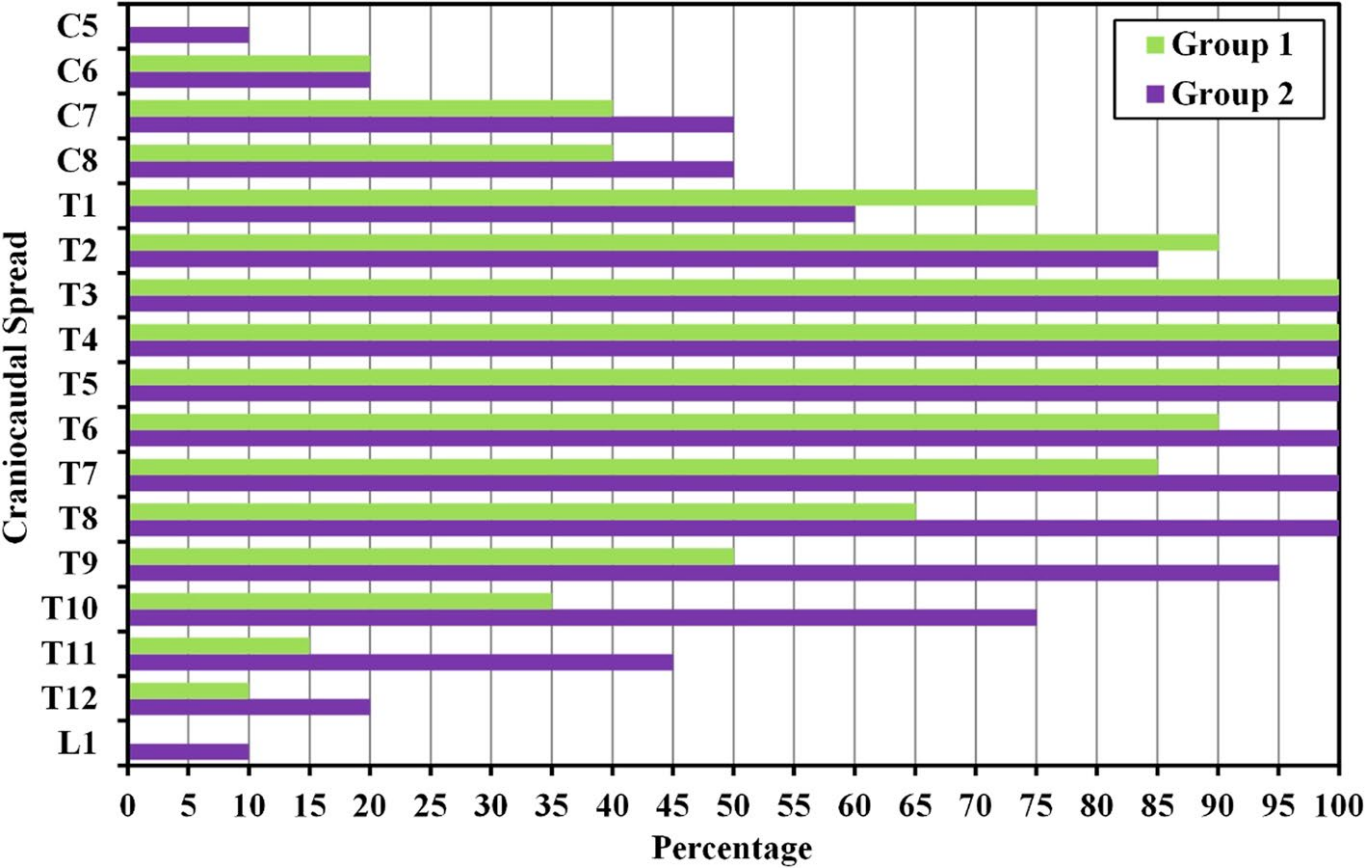
Comparison among the two intervention groups based on CranioCaudal dye spread

**Fig. 5:**
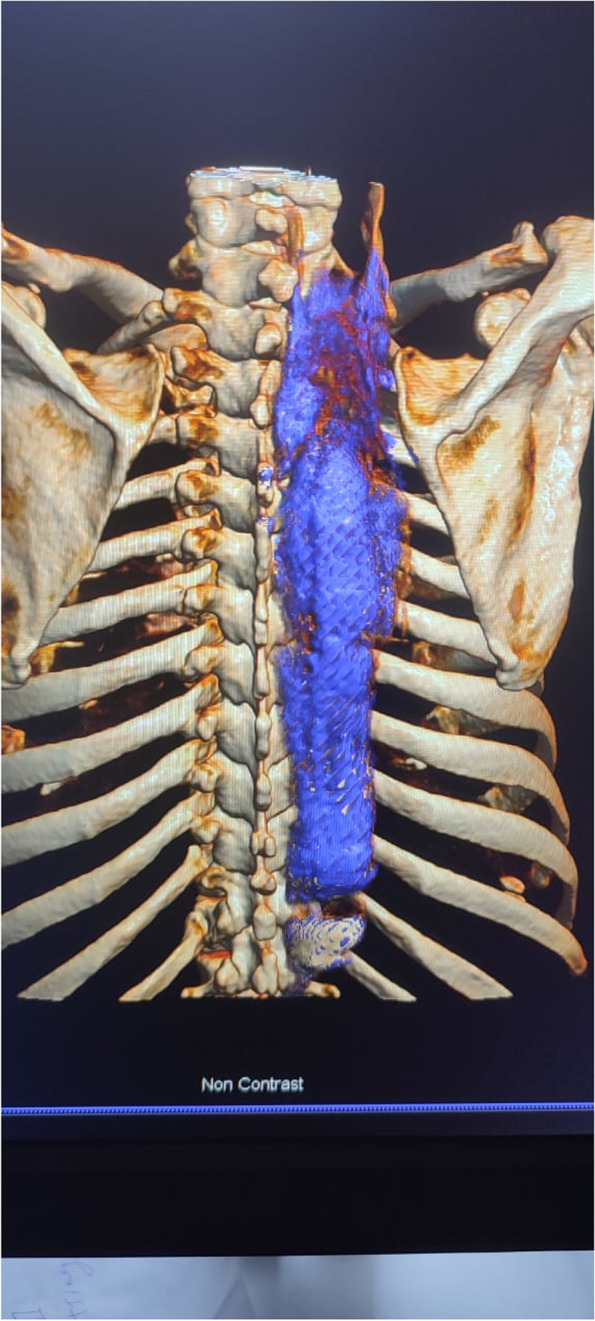
3 D reconstruction by CT (postero-anterior view) of the craniocaudal spread of LA-dye mixture

The LA-dye mixture had extended further, from the site of injection at T4 vertebral level, in the high volume group than the standard volume group (11.20 ± 3.07 vs. 9.15 ± 2.54 vertebral levels, *p* = 0.027). (Table 2 and Figs. 4 and 5).

**Table 2:** Comparison among the two intervention groups based on CT spread

CT spread	Standard volume group (***n*** = 20)	High volume group (***n*** = 20)	Test of Sig.	***P***
**Craniocaudal**				
Mean ± SD.	9.15 ± 2.54	11.20 ± 3.07	t = 2.300^*^	0.027^*^
Median (Min. – Max.)	10.0 (4.0 – 14.0)	10.50 (7.0 – 17.0)		
**Paravertebral**	**6 (30.0%)**	**8 (40.0%)**	χ^2^ = 0.440	0.507
Mean ± SD.	1.0 ± 0.0	1.25 ± 0.46	t = 1.528	0.170
Median (Min. – Max.)	1.0 (1.0 – 1.0)	1.0 (1.0 – 2.0)		
**Epidural**	**0 (0.0%)**	**4 (20.0%)**	χ^2^ = 4.444	^FE^*p* = 0.106
Mean ± SD.	–	5.0 ± 1.15	–	–
Median (Min. – Max.)	–	5.0 (4.0 – 6.0)		
**Exiting Spinal nerve roots**	**0 (0.0%)**	**4 (20.0%)**	χ^2^ = 4.444	^FE^*p* = 0.106
Mean ± SD.	–	1.0 ± 0.0	–	–
Median (Min. – Max.)	–	1.0 (1.0 – 1.0)		
**Crossed midline**				
No	18 (90.0%)	18 (90.0%)	–	–
Yes	2 (10.0%)	2 (10.0%)		

χ^2^ Chi square test, *FE* Fisher Exact, *SD* Standard deviation, *t* Student t-test

p: *p* value for comparing among the two intervention groups

*: Statistically significant at *p* ≤ 0.05

**Fig. 6:**
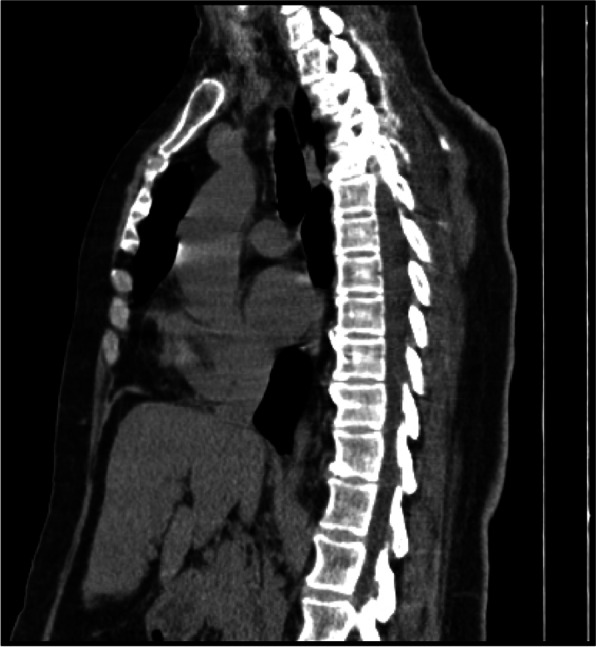
Sagittal CT scan demonstrating paravertebral spread of LA-dye mixture at T4-T5 (Right exit foramen)

Thirty and forty percent of cases showed spread to PV space in the standard volume ESPB and high volume ESPB groups, respectively and the difference is statistically insignificant (*p* = 0.507) (Table 2 and Fig. 6).

**Fig. 7:**
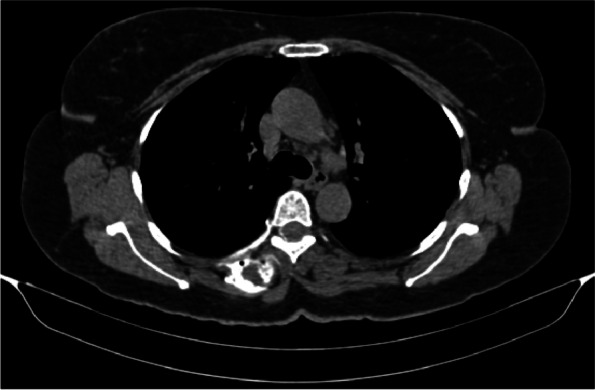
Axial CT scan demonstrating epidural spread of LA-dye mixture at T5 level

No spread to the epidural space or to the exiting spinal nerve roots had been observed in the standard volume ESPB group while the incidence was 20% in the high volume ESPB group, yet the difference is insignificant (*p* = 0.106). (Table 2 and Fig. 7).

**Fig. 8:**
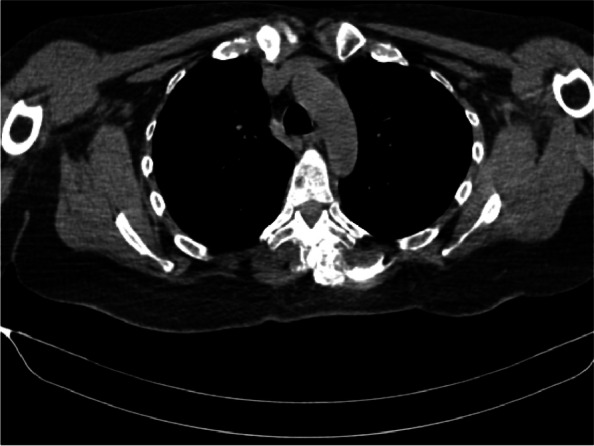
Axial CT scan showing accidental crossing of LA-dye mixture to the contralateral side at T4 level

Ten percent of the cases in each ESPB group showed dye in the contralateral side. (Fig. 8).

#### Clinical dermatomal assessment

**Fig. 9:**
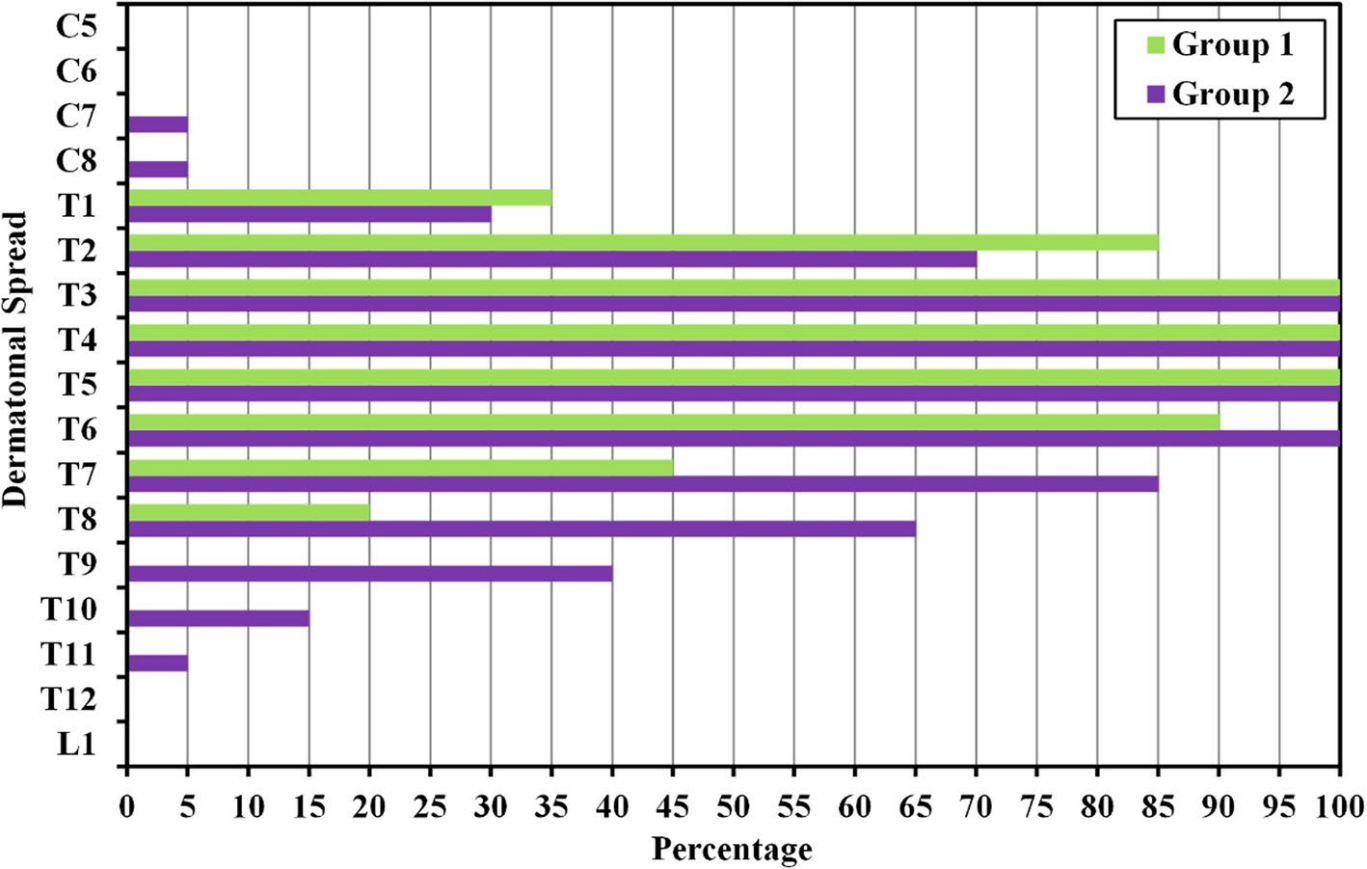
Comparison among the two intervention groups based on clinical dermatomal level achieved

Regarding the dermatomal level of the block, more dermatomes were covered in the high volume group relative to the standard volume group (7.20 ± 2.12 vs. 5.75 ± 1.37 dermatomes, *p* = 0.014). (Table 3 & Fig. 9).

**Table 3:** Comparison among the two intervention groups based on the dermatomal level achieved

Dermatomal level (dermatomes)	Standard volume (***n*** = 20)	High volume (***n*** = 20)	t	P
Mean ± SD.	5.75 ± 1.37	7.20 ± 2.12	2.570^*^	0.014^*^
Median (Min. – Max.)	6.0 (3.0 – 8.0)	7.0 (4.0 – 13.0)		

p: *p* value for comparing among the two intervention groups

*: Statistical significance at *p* ≤ 5%

#### Correlation

**Fig. 10:**
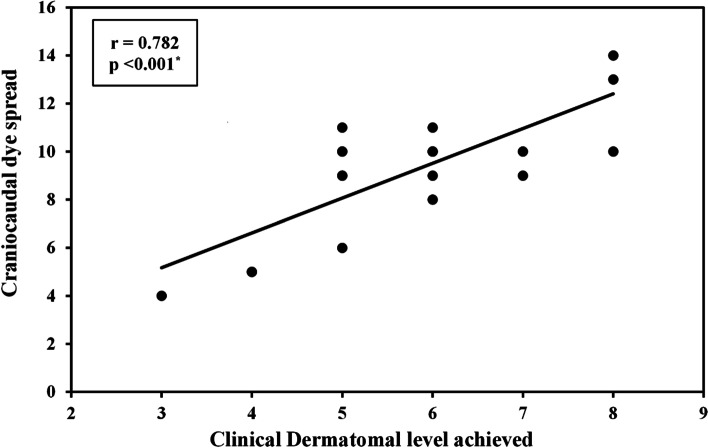
Correlation between craniocaudal dye spread and clinical dermatomal level achieved in the standard volume ESPB group

**Fig. 11:**
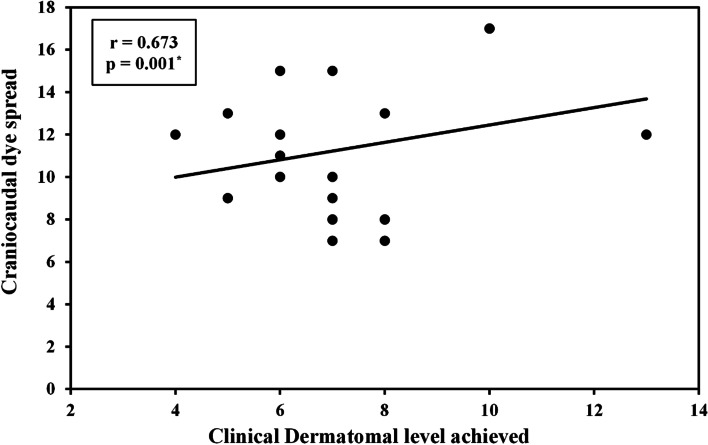
Correlation between craniocaudal dye spread and clinical dermatomal level achieved in the high volume ESPB group

There was a statistically significant strong correlation between Craniocaudal dye spread and clinical dermatomal level achieved in both the standard volume and high volume ESPB groups (r = 0.782, *p* < 0.001 and 0.673, *p* = 0.001). (Figs. 10 and 11).

### Sedation

**Fig. 12:**
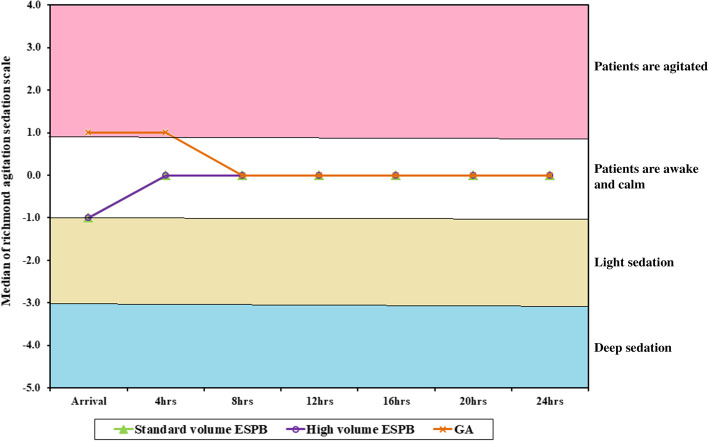
Comparison among the three groups based on Richmond agitation sedation scale

At arrival to the PACU, most of the cases in the standard volume and high volume ESPB groups were lightly sedated (median RASS = − 1 in both groups) while in the GA group most of the cases were agitated (median RASS = 1, *p* = 0.002). Four hours thereafter, the ESPB groups were awake and calm (median RASS = 0) and GA group cases were still agitated (median RASS = 1, *p* = 0.01). From 8 hours postoperative till the end of observation time, most of the cases overall were awake and calm. (Fig. 12).

### Patient satisfaction

Regarding patient satisfaction with the overall analgesic technique, both ESPB groups showed statistically higher level of satisfaction than the GA only group (*p* < 0.001), yet the volume injected made no difference. (Table 4).

**Table 4:** Comparison among the three groups based on patient satisfaction

Patient Satisfaction	Standard volume group (***n*** = 20)		High volume group (***n*** = 20)		GA only group (***n*** = 20)		χ^**2**^	^**MC**^p
	No.	%	No.	%	No.	%		
Very dissatisfied	0	0.0	0	0.0	0	0.0	41.125^*^	< 0.001^*^
Dissatisfied	0	0.0	0	0.0	6	30.0		
Neutral	0	0.0	0	0.0	10	50.0		
Satisfied	6	30.0	4	20.0	2	10.0		
Very Satisfied	14	70.0	16	80.0	2	10.0		

χ^2^ Chi square test, *MC* Monte Carlo

p: *p* value for comparing among the three groups

*: Statistically significant at *p* ≤ 0.05

There was neither correlation between Craniocaudal dye spread and VAS nor the Craniocaudal dye spread and patient satisfaction in both ESPB groups. (Tables 5 and 6).

**Table 5:** Correlation between Craniocaudal dye spread and VAS in each intervention group

VAS	Craniocaudal dye spread			
	Standard volume group (***n*** = 20)		High volume group (***n*** = 20)	
	R	p	R	p
**1 hr**	−0.493	0.027^*^	−0.167	0.481
**2 hrs.**	−0.173	0.465	−0.015	0.949
**3 hrs.**	−0.009	0.969	0.162	0.496
**4 hrs.**	0.034	0.887	0.299	0.200
**8 hrs.**	0.038	0.873	−0.034	0.888
**12 hrs.**	−0.112	0.638	−0.338	0.145
**16 hrs.**	−0.328	0.158	−0.329	0.157
**20 hrs.**	0.002	0.994	−0.208	0.379
**24 hrs.**	0.002	0.993	−0.291	0.213

r: Pearson coefficient

*: Statistically significant at *p* ≤ 0.05

**Table 6:** Correlation between Craniocaudal dye spread and patient satisfaction with the overall analgesic technique in each ESPB group

	Standard volume group (***n*** = 20)		High volume group (***n*** = 20)	
	R	p	R	p
**Craniocaudal dye spread vs. patient satisfaction**	−0.357	0.122	−0.008	0.972

## Discussion

This is the first prospective, randomized, triple-blind study using variable LA volumes and concentrations in ESPB in breast cancer procedures. Our findings show that a large LA volume in ESPB, when giving the same dose, has no effect on analgesic effectiveness; however, as compared to GA only without ESPB, it shows a marked improvement. On the other hand, the LA had spread radiologically further and blocked more dermatomes clinically in the high volume group yet, didn’t affect the VAS or patient satisfaction.

We have doubled the volume and halved the concentration of LA from the standard volume to the high volume ESPB groups, so we used the same dose of 50 mg bupivacaine in both ESPB groups. Altiparmak and his colleagues [18] injected fixed volume of 20 ml but with variable concentrations of 0.375 and 0.25%, yielding 75 mg and 50 mg bupivacaine in each group. The differences in outcomes between the two investigations could be explained by the aforementioned. In our investigation, there was no change in VAS ratings between the two intervention groups, however Altiparmak’s study found that the high dose group had much more decrease in NRS (Numeric rating scale) and the need for postoperative opioids. As a result, rather than the volume or concentration, the analgesic effectiveness of ESPB may be governed by the LA dosage.

The optimal volume of ESPB is still up for dispute; however studies on pediatric populations have focused on it. Tulgar and colleagues [13] offer a dosage of 0.5 ml/kg, whereas Govender et al. [19], advocate a dose of 0.1 ml/kg/dermatome. The latter study’s findings cannot be generalized because it was done on cadaveric preterm infants. In our study, the relationship between volume injected and the spread is not linear as doubling the volume from 20 to 40 ml had resulted in 22% increase in the craniocaudal spread (from 9.15 to 11.2 vertebral levels).

Also, Zhang et al. [20], conducted a meta-analysis of 11 RCTs comprising 679 patients and found that ESPB for breast cancer procedures is a beneficial analgesic treatment and markedly attenuates pain severity during the first 24 postoperative hours, when compared to GA alone. According to a meta-analysis of 13 RCTs involving 861 patients, Leong and his team [21] found that ESPB reduces pain scores for 24 hours after breast surgeries, and its effectiveness is equivalent to paravertebral block.

The anatomical studies revealed that the injectate distribution in ESPB follows the PV block (PVB) pathway, which involves both the ventral and dorsal spinal rami and has a mechanism similar to PVB, as well as the lateral pathway, which involves the lateral cutaneous branch and small branches of intercostal nerves and has a mechanism similar to that of interfascial plane blocks [22].

In the current study, high injection volume resulted in a better craniocaudal spread. Epidural and paravertebral spread had not been affected by volume and the difference is statistically insignificant between the two groups. The pattern of distribution and craniocaudal spread of the LA-dye mixture in the standard volume ESPB group in the current study resembles the study of Ivanusic et al., [15] though the latter is a cadaveric one. The anatomical study by Azevedo and his colleagues [23], comparing different injection volumes, disagrees with the current study and had proved that lumbar ESPB have a volume dependent spread. Twenty mL injections had no anterior spread while, higher volumes (30 and 40 mL) had spread anteriorly, reaching the lumbar plexus.

The block application site (e.g., upper thoracic, lower thoracic, or lumbar) is crucial and has a considerable impact on clinical findings of the ESPB. Lumbar ESPB varies from thoracic ESPB in terms of technique and anatomical structure. The transverse process of the thoracic vertebra is 2–3 cm lateral, while that of the lumbar vertebra can be 4–6 cm lateral so in lumbar ESPBs, a large paravertebral dispersion of injected fluid is unlikely. Thoracic ESPB has a clinical impact comparable to an extended paravertebral block, whereas lumbar ESPB has a clinical effect similar to a lumbar plexus block [24].

By looking at the spread to the PV space and focusing on cadaveric studies that applied ESPB in upper thoracic region, we found conflicting outcomes. Spread to the PV space had been demonstrated in eight cadaveric studies [16, 19, 25–30] and denied in three studies [15, 31, 32]. The volume used in these studies was 20 ml of dye except shibata et al. [32] (15 ml), Choi et al. [28] (10 & 30 ml), and Govender et al. [19] (Cadaveric preterm neonates). The current study confirmed the spread to the paravertebral space in 30% of cases in the standard volume ESPB group and 40% of cases in the high volume ESPB group. The 10% difference between the 2 groups is statistically insignificant, so the spread to the PV space may depend on other factors beside the volume of LA injected.

The endoscopic and anatomic study by Choi et al. [28] had revealed a marked difference between 10 ml and 30 ml volumes regarding spread to PV space. Thirty ml is better than 10 ml but not the 20 ml used in their previous study [16]. Although PV spread increased in a volume-dependent way after ESPB, the rise was variable and not dramatic. The degree of injectate spread to the back muscles and fascial layers seemed to be largely enhanced when the injectate volume for the ESPB increased, compared to the extent of PV spread. To recapitulate, ESPB may cause injectate spread to a larger area of the thoracic back region as a function of injection volume, however the degree of PV spread may not be considerably increased by increasing injection volume beyond 20 ml at a single level [28].

PV craniocaudal spreading [33] from a single injection site in a conventional USG PVB with 20 ml of injectate might reach up to 3 to 4 vertebral levels. As a result, compared to traditional PVB, PV craniocaudal spreading following ESPB appears to be significantly reduced [34, 35].

The current study showed the spread to the epidural space in 20% of cases in the high volume ESPB group and not to any case in the standard volume ESPB group. The results of other studies are variable; epidural spread had been confirmed in four cadaveric studies [19, 28–30], and contradicted in another four studies [15, 25, 26, 31]. So, the LA volume injected in the ESP is not the sole factor affecting the spread to the epidural space.

The dye distribution in a cadaveric model may differ from that seen in the living. Different tissues tension, muscle tone, body temperature, solution density, and variations in intra-abdominal pressure generated by breathing, not present in cadavers, are all anatomical factors that may affect the spread. Furthermore, the LA distribution may differ from that of the dye solution [23].

Among the analgesic mechanisms of ESPB are LA spread to the PV and epidural spaces; Systemic absorption of LA increasing its plasma concentration; LA immunomodulation; and LA effect on the thoracolumbar fascia. Clinical, physiological cadaveric, veterinary, and biomechanical laboratory research all point to a direct action of LA on neuronal structures in the fasciae deep to ES muscle and nearby tissues as the most probable fundamental mechanism [17].

In the current study, the dermatomal coverage of the block is better in the high volume group, 40 ml as opposed to the standard volume of 20 ml. The 40 ml resulted in a mean dermatomal block of 7.2 dermatomes and the 20 ml resulted in a mean dermatomal block of 5.75 dermatomes. Doubling the volume of LA had led to only 25% increase in the dermatomal coverage so the relationship between the LA volume and the number of dermatomes blocked is not a linear relationship. Barrios and his coworkers [36], evaluated the sensory mapping of ESPB and they concluded that using a single injection of 20 mL of 0.5% plain bupivacaine at the mid-thoracic level results in a mean dermatomal spread of 9 dermatomes (range, 8-11). The difference between this result and that of the current study may be due to the different concentration used or more probably due to the time factor and the modality of sensory assessment. In the current study, the sensory block has been assessed by the presence of hypoesthesia along the mid-clavicular line 15 minutes after the block using iced water-soaked piece of cotton, while Barrios has evaluated the sensory block 60 minutes after completion of ESPB by change in feeling to both pinprick and cold methods. Time is an important factor in the gradual spread of LA. The progressive diffusion of LA clinically over hours rather than minutes is a crucial element in dynamic pressures throughout tissue planes and compartments in the living [17, 32]. The mean volume of LA required per dermatome in ESPB is 2.2 ml (range from 1.81 to 2.5 ml) according to Barrios et al. [36], and 3.4 mL (range from 2.5 to 6.6 mL) according to Cassai et al. [37]. The latter is a case series study and the dermatomal spread variable was not prospectively controlled while Barrios et al. [36], is a prospective cohort. The current study revealed a discrepancy between the 2 groups as in the standard volume group 3.4 ml of LA is required to block a dermatome and 5.5 ml is required in the high volume group.

ESPB is non-inferior to PVB in terms of controlling post-mastectomy pain [33]. But even the PVB can’t provide a full intraoperative or postoperative analgesia as it can provide analgesia to the 4 quadrants of the breast but not to the infra-clavicular region, which needs LA infiltration. Pectoral nerves (PECS) II block provides analgesia to the upper outer quadrant and Serratus plane block (SPB) provides analgesia to the lower outer quadrant. Parasternal block and transversus thoracis muscle plane block provides analgesia to the inner quadrants. Combining fascial plane blocks is recommended for complete intraoperative and postoperative analgesia for oncological breast surgerie s[38]..

ESPB can substitute the epidural injection in selected cases as a less invasive & safer procedure with a similar effect [39]. It reduces the risk of epidural side effects (motor block [13.4%], dural puncture [1.2%], epidural hematoma [0.02%], post-puncture headache [0.14%], and postoperative neurological deficit [1.2%]) [40]. Furthermore, it can be performed in patients on anticoagulants [36].

Evaluating the dye spread by CT, we found 2 cases (10%) in which the dye crossed the midline in both the standard volume and high volume groups, so this phenomenon may not be volume dependent. In concordance to this, Tulgar et al. [41] reported a case in which bilateral sensory blockade had occurred after unilateral ESPB.

RASS was the method chosen to determine the difference of the level of sedation or agitation between the groups in the current study and the results proved that ESPB resulted in less agitation compared to GA alone, yet the difference in volume of the block made no difference in either sedation or agitation. In agreement with the current study, Unal et al. [42] reported higher frequency of agitation in the control group compared to ESPB group. Also, Shim et al. [43] proved that ESPB successfully attenuated the degree of postoperative emergence agitation. On the converse, Elsabeeny and his colleagues [44] reported higher level of sedation in the control group than ESPB group. The scales used for assessment may explain the difference in these results. Riker sedation agitation scale was used in Unal et al. [42], and Shim et al. [43], studies, while Ramsay sedation scale was used in Elsabeeny’s study [44]. The scales are nearly similar in terms of the inter-rater reliability, yet they are different in the design and construct validity. Apart from the different scales used in these studies, another point of explanation is that ESPB can decrease agitation due to better analgesia and it can also decrease sedation due to less need for opioids.

The current study demonstrated that ESPB results in a higher level of satisfaction among patients compared to GA alone, yet the difference in volume made no difference. Park et al. [45], in consistence with this results, proved that ESPB had improved patient satisfaction after mastectomy without drawbacks. Also, Yao et al. [46], showed that the quality of recovery and patient satisfaction had been markedly enhanced after application of thoracic ESPB. Furthermore, meta-analysis by Oh and his colleagues [47] demonstrated that using ESPB had improved patient satisfaction and recovery.

The current study may have some limitations. In the control group, pre-emptive analgesia was not adequate and this may explain that many patients in this group were agitated for 8 postoperative hours. For future studies, adequate multimodal and pre-emptive analgesia is recommended in the control group when it comes to the generalizability of the findings.

The interval between blockade and CT scanning, 15 minutes, may be too short to allow adequate and optimum LA spread. A key factor in the dynamic pressures across tissue planes and compartments in the living is the clinically observed gradual and progressive diffusion of LA over hours as opposed to minutes.

The term “standard volume ESP” may be mısguiding as there is no standard volume set for ESP so far. In the current study it simply means 20 ml, which is utilized in most studies and which is the contrast of “high volume ESPB” group.

## Conclusion

Preoperative ESPB is an excellent analgesic modality that should be considered in breast cancer surgeries. It can also attenuate both postoperative agitation and sedation. Doubling the injectate volume enhances the craniocaudal spreading and may be useful for surgeries requiring multiple dermatomes. However, larger volume has no effect on analgesic efficacy or patient satisfaction as there is no further spread to the paravertebral space, epidural space or spinal nerve rami.

## Data Availability

The trial was prospectively registered at ClinicalTrials.gov (NCT04796363; registration date: 12/3/2021). The data used to support the findings of this study are available from the corresponding author upon request.

## Abbreviations

ALND: Axillary lymph node dissection
ANOVA: Analysis of variance
ASA: American society of Anesthesiology
C_max_: Peak plasma concentration
CONSORT: Consolidated Standards of Reporting Trials
CT: Computed tomography
DICOM: Digital Imaging and Communications in Medicine
ES: Erector Spinae
ESP: Erector Spinae Plane
ESPB: Erector Spinae Plane Block
GA: General anesthesia
IRB: Institutional Review Board
IV: Intravenous
LA: Local anesthetic
LAST: Local anesthetic systemic toxicity
LN: Lymph node
MRM: Modified radical mastectomy
NRS: Numeric rating scale
PACU: Post-anesthetic care unit
PCA: Patient controlled analgesia
PECS: Pectoral nerves
PONV: Post-operative nausea or vomiting
PV: Paravertebral
PVB: Paravertebral block
RASS: Richmond agitation sedation scale
SPB: Serratus plane block
T4: The fourth thoracic vertebra
TPVB: Thoracic paravertebral block
USG: Ultrasound guided
VAS: Visual analogue scale
